# Palatoglossus Muscle and T4 Category in the Eighth Edition of TNM Staging System for OPSCC

**DOI:** 10.1002/ohn.957

**Published:** 2024-08-27

**Authors:** Giancarlo Tirelli, Nicoletta Gardenal, Jerry Polesel, Jasmina De Groodt, Erik Radin, Fabiola Giudici, Laura Iandolo, Simone Zucchini, Egidio Sia, Paolo Boscolo‐Rizzo

**Affiliations:** ^1^ Department of Medical, Surgical and Health Sciences, Section of Otolaryngology University of Trieste Trieste Italy; ^2^ Unit of Cancer Epidemiology, Centro di Riferimento Oncologico di Aviano (CRO) Istituto di Ricovero e Cura a Carattere Scientifico (IRCCS) Aviano Italy; ^3^ Department of Medical, Surgical and Health Sciences, Section of Radiology University of Trieste Trieste Italy

**Keywords:** human papillomavirus, oropharyngeal cancer, p16, palatoglossus muscle, surgery, TNM staging system

## Abstract

**Objective:**

The present study challenges the appropriateness of considering invasion of the palatoglossus muscle (PGM) as a criterion for staging oropharyngeal squamous cell carcinoma (OPSCC) as T4.

**Study Design:**

Retrospective observational study.

**Setting:**

Tertiary University Hospital.

**Methods:**

This retrospective study included nonmetastatic OPSCC patients treated with curative intent at the University of Trieste, Italy from 2015 to 2021. Patients were categorized into 4 groups: (1) tumors classified as T1‐T2 by both International Cancer Control (UICC) and American Joint Committee on Cancer (AJCC)‐TNM; (2) T1‐T2 tumors upgraded to T4 solely by UICC due to oropharyngeal PGM infiltration; (3) T1‐T2 tumors upgraded to T4 by both UICC and AJCC due to oral PGM infiltration; (4) tumors classified as T3‐T4 by both UICC and AJCC. Kaplan‐Meier analysis estimated overall survival (OS) and disease‐free survival (DFS). Multivariable Cox models, adjusted for clinical factors, assessed the impact of palatoglossus invasion on outcomes over 5 years.

**Results:**

A total of 121 consecutive patients with primary OPSCC were included (median [interquartile range] age 65 years [58‐74]; 63% male). While patients with upgraded T4 category due to infiltration of the oral portion of the PGM exhibited a prognosis superimposable on that of other patients with advanced stage disease, those with upgraded T4 category due to infiltration of the oropharyngeal portion of the PGM displayed OS and DFS comparable to T1‐T2 patients.

**Conclusion:**

Our findings highlight that invasion of the oropharyngeal portion of the PGM may not be a suitable criterion for staging OPSCC as T4. Further research involving larger and independent patient cohorts is strongly encouraged to corroborate these observations.

The TNM classification system for head and neck cancer, as outlined by the American Joint Committee on Cancer (AJCC) and the Union for International Cancer Control (UICC), serves as a cornerstone in the staging and management of head and neck cancer. In the realm of oropharyngeal squamous cell carcinoma (OPSCC), the distinction between T categories, particularly the designation of a tumor as moderately advanced (T4a for p16‐negative tumors, T4 for p16‐positive tumors), also hinges upon the invasion of extrinsic tongue musculature (genioglossus, hyoglossus, styloglossus, and palatoglossus muscles [PGMs]).^1^, ^2^

The inclusion of the PGM—a superficial muscle with minimal connection to the deeper musculature of the tongue and primarily located a few millimeters from the mucosa in the anterior tonsillar pillar—raises questions regarding its impact on cancer staging.^3^, ^4^

Importantly, the TNM staging system is not just a descriptive tool but also has prognostic implications and accurate staging is also crucial for determining the appropriate therapeutic approach, as treatment modalities (surgery, radiation therapy, chemotherapy, or a combination thereof) are often guided by the stage of the disease.^5^ Therefore, it is essential to consider the potential implications of over‐staging or under‐staging on patient outcomes and quality of life, as more aggressive treatments may be associated with increased morbidity and adverse effects.^6^

Significantly, the AJCC and the UICC classifications present minor differences in their criteria, particularly regarding the infiltration of the PGM. The AJCC classification^1^ specifies that for a tumor to be classified at a higher T category, it must infiltrate at the level of the oral floor. Conversely, the UICC classification^2^ does not prioritize the level of the muscle infiltration thus including the infiltration of the oropharyngeal portion of the PGM as a criterion for categorizing a tumor as T4. The proximity of this muscle to the mucosal surface means that even small tumors originating from the soft palate or tonsil and invading the anterior tonsillar pillar could classify a cancer as T4, potentially overestimating its aggressiveness.

Considering these aspects, the current study was designed to assess the overall survival (OS) and disease‐free survival (DFS) for patients with OPSCC categorized as T4, exclusively based on the criterion of PGM invasion.

## Materials and Methods

This is a retrospective study including patients diagnosed with nonmetastatic (M0) OPSCC who underwent curative‐intent treatment at the at the Clinic of Otolaryngology‐Head and Neck Surgery, University of Trieste, Trieste, Italy, from January 2015 to January 2021. Patients received radiochemotherapy or surgery ± adjuvant therapy based on National Comprehensive Cancer Network guidelines and patient preference, and were followed‐up until December 31, 2023. The study received approval from the ethics committee of the University of Trieste (ethic vote: 137/2024).

All patients underwent staging through both contrast‐enhanced computed tomography (CT) and magnetic resonance imaging (MRI) scans, with Positron emission tomography‐CT performed in advanced stages to rule out distant metastases. Two radiologists experienced in OPSCC imaging thoroughly examined all CT and MRI imaging studies to evaluate the presence of palatoglossus invasion. The PGM was divided into oropharyngeal and oral portions by an imaginary line extending through the dorsum at the base of the tongue in coronal scans (Supplemental Figure S1, available online). Any disagreements between the radiologists were resolved after joint re‐evaluation of the case.

To evaluate whether the inclusion of PG muscle involvement identify patients at higher risk of death, we created a new T category, starting from a new T staging that omitted palatoglossus invasion as a criterion for T4 staging, namely “T category without PGM” (TwPGM). Patients were categorized into 4 groups: (1) patients with tumors consistently classified as T1‐T2 according to both UICC and AJCC‐TNM guidelines; (2) patients with T1‐T2 tumors upgraded to T4 based solely on the UICC criteria, which is specific to the infiltration of the oropharyngeal portion of the PGM; (3) patients with T1‐T2 tumors upgraded to T4 under both UICC and AJCC criteria, due to infiltration of the oral portion of the PGM; and (4) patients with tumors classified as T3‐T4 based on other criteria than PGM invasion.

For each patient, the time at risk was calculated from treatment end to the event of interest or follow‐up end. The event of interest was defined as death for OS and as recurrence or death for DFS. Survival probabilities for OS and DFS were estimated by means of the Kaplan‐Meier method, and differences across strata were evaluated through the log‐rank test. Hazard ratios (HRs) and corresponding confidence intervals (CIs) for death were estimated through multivariable Cox proportional hazards model, adjusting for recognized clinical predictors of OS or DFS (namely, gender age, cancer subsite, human papillomavirus [HPV] status, and treatment). Analyses were truncated at 5 years after the end of treatment.

## Results

A total of 121 consecutive patients with primary OPSCC were included, with a median (range) age of 65 years (*Q*_1_‐*Q*_3_: 58‐74 years); 62.8% were men (Table 1). Most of cases arose from the tonsillar complex (58.7%). Overall, 54 cases were HPV‐driven (50.4%), based on concomitant p16^INK4a^ overexpression and HPV DNA positivity. Sixty‐four patients (52.9%) underwent up‐front surgery while 57 (47.1%) received up‐front (chemo)‐radiotherapy.

**Table 1:** Sociodemographic and Clinical Characteristics

	n	(%)
Gender		
Male	76	(62.8)
Female	45	(37.2)
Age, y		
Median (*Q*_1_‐*Q*_3_)	65	(58‐74)
Subsite		
Tonsil	71	(58.7)
Base of tongue	33	(27.3)
Soft palate	11	(9.1)
Posterior wall	6	(5.0)
HPV‐driven		
No	66	(54.5)
Yes	55	(45.5)
Treatment		
Surgery	23	(19.0)
Radio‐/chemotherapy	57	(47.1)
Surgery + radio‐/chemotherapy	41	(33.9)
TwPGM category		
T1	27	(22.3)
T2	24	(19.8)
T3	13	(10.7)
T4	57	(47.1)
N category		
N0	27	(22.3)
N1	37	(30.6)
N2	48	(39.7)
N3	9	(7.4)

Abbreviation: HPV, human papillomavirus; TwPGM, T category without palatoglossus muscles invasion.

When evaluating the infiltration of PGM, agreement between the 2 radiologists occurred in 87.6% of cases. In 15 cases (12.4%) where there was initial disagreement, consensus was reached after joint re‐evaluation

**Figure 1:**
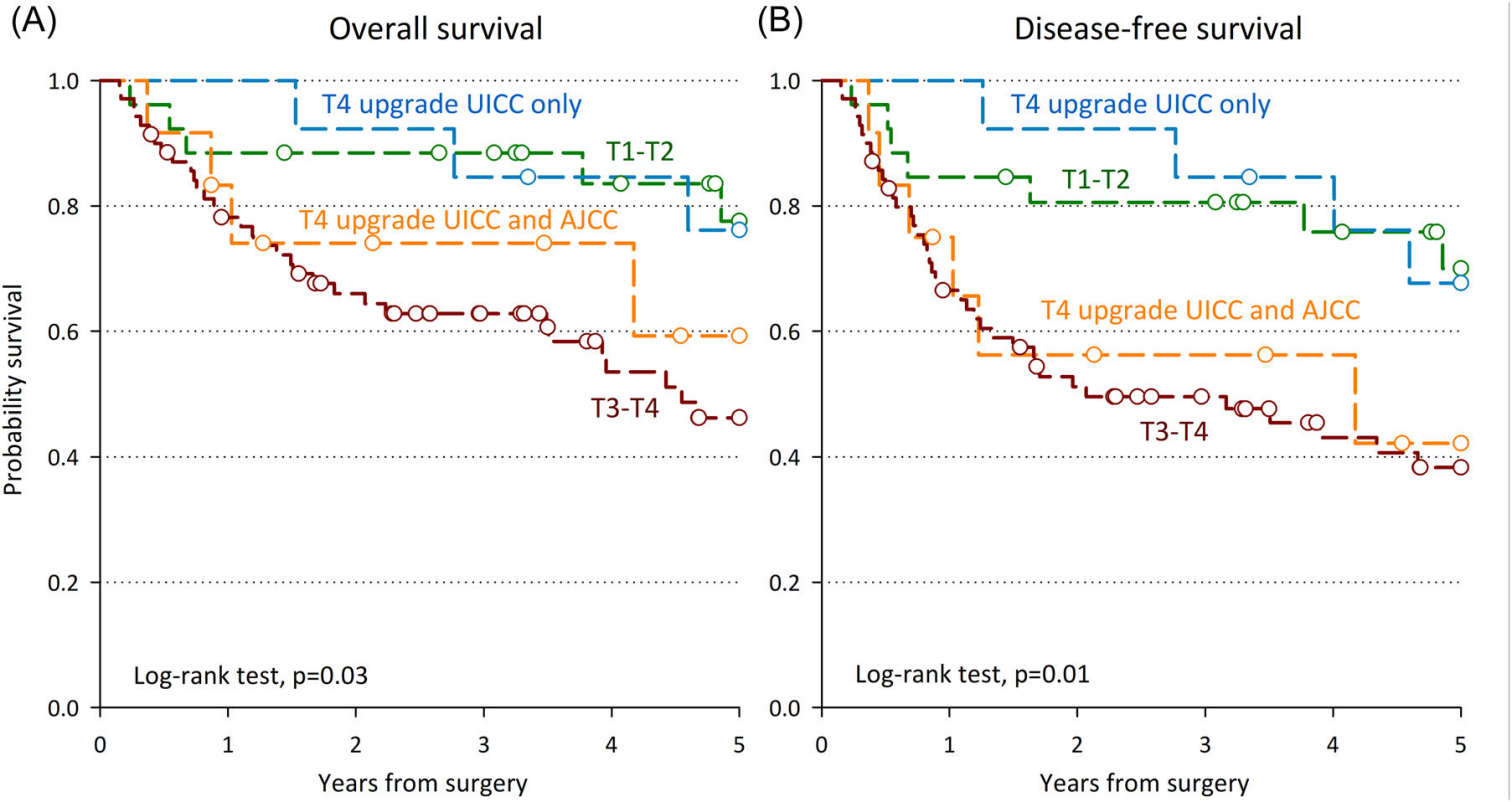
Overall survival (A) and disease‐free survival (B) by T category classification. Blue: T4‐upstaged due to oropharyngeal palatoglossus invasion. Orange: T4‐upstaged due to oral palatoglossus invasion. Green: early T1‐T2 stage. Red: advanced T3‐T4 stage.

Fifty‐one patients were T1‐T2 according to TwPGM (Table 1). Among them when considering the invasion of the PGM, 25 cases (49.0%) were upgraded to T4 category; these include 13 cases classified according to the AJCC criteria, due to the invasion of the oral portion of the muscle, and an additional 12 cases as per UICC staging criteria, due to the invasion of the oropharyngeal portion of the muscle (Table 2). Notably, the 12 patients with upgraded T4 category due to the infiltration of the oropharyngeal portion of the PGM showed an OS and DFS as good as T1‐T2 patients (Figure 1A and B). Conversely, those with upgraded T4 category due to the infiltration of the oral portion of the PGM showed a prognosis superimposable to other patients with advanced T category. After accounting for gender, age, subsite, HPV‐status, and treatment, patients with upgraded T4 category due to the infiltration of the oral portion of the PGM had a 4.9‐fold higher risk (95% CI: 1.4‐16.5) of disease recurrence or death than T1‐T2 patients, as high as T3‐T4 patients (HR = 3.0; 95% CI: 1.1‐7.8) (Table 3).

**Table 2:** Variation in T Category Using UICC and AJCC in Comparison to T category Without Palatoglossus Muscle Evaluation (TwPGM)

	Variation of UICC vs TwPGM			
	Confirmed T1‐T2	Upgrade to T4	Confirmed T3‐T4	Total
Variation of AJCC vs TwPGM				
Confirmed T1‐T2	26	12	01	38
Upgraded to T4	0	13	0	13
Confirmed T3‐T4	0	0	70	70
Total	26	25	70	121

Abbreviations: AJCC, American Joint Committee on Cancer; TwPGM, T category without palatoglossus muscles invasion; UICC, Union for International Cancer Control.

**Table 3:** HRs of Recurrence or Death and Corresponding 95% CIs According to Recognized Clinical Predictors and T Staging

	Patients	Recurrences or Deaths	Univariate HR (95% CI)	Multivariable^a^ HR (95% CI)
Gender				
Male	76	45	Reference	Reference
Female	45	11	0.33 (0.17‐0.64)	0.40 (0.20‐0.82)
Age, y			1.02 (1.00‐1.05)	1.02 (1.00‐1.05)
Subsite				
Tonsil	71	29	Reference	Reference
Base of tongue	33	19	1.74 (0.98‐3.11)	1.71 (0.91‐3.24)
Soft palate	11	5	0.99 (0.38‐2.55)	0.58 (0.21‐1.60)
Posterior wall	6	3	0.90 (0.27‐2.96)	0.47 (0.13‐1.71)
HPV‐driven				
No	66	34	Reference	Reference
Yes	55	22	0.79 (0.46‐1.36)	0.67 (0.36‐1.25)
Treatment				
Surgery	23	13	Reference	Reference
RCT	57	30	0.91 (0.48‐1.75)	0.49 (0.21‐1.11)
Surgery + RCT	41	13	0.41 (0.19‐0.89)	0.35 (0.15‐0.80)
T staging				
T1‐T2	26	7	Reference	Reference
T4 upgrade UICC	12	6	1.00 (0.29‐3.41)	0.80 (0.22‐2.86)
T4 upgrade UICC/AJCC	13	4	2.56 (0.86‐7.66)	4.86 (1.43‐16.54)
T3‐T4	70	39	2.91 (1.30‐6.53)	2.98 (1.13‐7.82)

Abbreviations: AJCC, American Joint Committee on Cancer; CI confidence interval; HPV, human papillomavirus; HR, hazard ratio; RCT, radiochemotherapy; UICC, Union for International Cancer Control.

^a^ Adjusted for all variable in the table.

## Discussion

The T4 classification according to current guidelines has profound clinical implications in the treatment of oropharyngeal carcinoma.^5^ This study raises critical questions about the validity of categorizing every form of invasion of the PGM as T4. The present study evaluated the prognostic impact of PGM invasion in OPSCC, highlighting differences based on the oral versus oropharyngeal portion involved. While infiltration of the oral portion of the PGM was associated with worse OS and DFS, on par with T3‐T4 disease, oropharyngeal PGM invasion had outcomes similar to T1‐T2 disease.

In defining a T4 stage, the involvement of extrinsic tongue muscles is considered as critical as the involvement of structures such as the larynx, hard palate, mandible, and medial pterygoid. Among these muscles, the PGM stands out as a small muscular fasciculus covered only by mucosa. It originates from the palatine aponeurosis of the soft palate, where it connects with the muscle on the opposite side. The PGM descends and moves laterally in front of the palatine tonsil, forming the anterior tonsillar pillar and inserting into the side of the tongue. Consequently, small tumors originating from the soft palate and tonsil can easily infiltrate the anterior tonsillar pillar by just a few millimeters due to their proximity.^7^ Additionally, from a surgical standpoint, the resection of the anterior tonsillar pillar is incorporated into the surgical procedure for accessing a tumor confined to the palatine tonsil.^8^ This removal neither extends the duration of the procedure nor introduces any additional complications.^9^

Unlike its upper portion, in the distal part, the PGM inserts deeply into the posterior two‐thirds of the tongue. In 1976, Du Brul hypothesized that the PGM extends toward the midline of the tongue, intermingling with the transverse muscles and creating a sphincter with the surrounding intrinsic muscle.^10^ The portion of the PGM infiltrated by the tumor could therefore have a different prognostic significance.

Our findings underscore the potential limitations of current staging paradigms. The UICC's uniform classification of any PGM invasion as T4 may overestimate the severity of oropharyngeal PGM involvement.^2^ On the other hand, the AJCC's distinction between oral and oropharyngeal PGM invasion appears to better reflect prognosis.^1^ The rationale for considering oral PGM invasion as a higher T category seems reasonable, as it could enable wider tumor spread given the muscle's role as an extrinsic tongue muscle. However, the oropharyngeal portion of the PGM lies very close to the mucosal surface, so its invasion may not necessarily indicate more advanced disease.^3^

These results align with previous radiological studies that highlighted a flaw in the AJCC staging system regarding extrinsic muscle involvement in oral cavity cancers. One study demonstrated through MRI measurements that even thin, superficial tumors can involve extrinsic muscles due to their proximity to the mucosal surface, suggesting this criterion alone may not accurately reflect true disease extent.^7^ A more recent cadaveric study examines the topography of the extrinsic tongue muscles through dissections to clarify whether involvement of these muscles in oropharyngeal cancers should systematically be classified as T4a tumors.^11^ The dissections revealed that the extrinsic tongue muscles (palatoglossus, styloglossus, hyoglossus) are located peripherally, while the genioglossus is the only deep extrinsic muscle with fibers extending to the tongue periphery and under the mucous membrane. Therefore, a T1 tumor invading the mucosa can affect the extrinsic muscles without being a T4a tumor. The authors argue that the term “deep extrinsic muscles of the tongue” is inaccurate and can lead to errors in staging oropharyngeal cancers based on a misunderstanding of tongue muscle anatomy.

Current literature lacks evidence to suggest that PGM involvement negatively impacts the prognosis of oropharyngeal carcinoma. In contrast, studies have revealed that while the involvement of the posterior pillar in oropharyngeal malignancies does correlate with local recurrence rates, the same cannot be said for the involvement of the anterior pillar.^12^

Limitations of this study include its retrospective nature at a single institution, which restricts broader generalization. Larger, prospective studies with standardized imaging review are needed to validate and extend our findings. The relatively small sample size limited certain subgroup analyses and may have compromised statistical power. Furthermore, the retrospective evaluation of imaging studies to assess PGM invasion is another limitation. A prospective study with predefined criteria and centralized radiologic review could enhance the accuracy and consistency of assessments regarding muscle involvement. Additionally, the study population underwent diverse treatment modalities such as surgery, radiation, and chemotherapy in various combinations. This diversity precluded a thorough evaluation of how different treatments affect outcomes in relation to palatoglossus status. A larger sample size, especially with sufficient representation across different treatment subgroups, would facilitate a more comprehensive assessment of this aspect. Finally, the initial disagreement between radiologists in some cases highlights the potential variability in assessing PGM involvement, which represents another limitation of using PGM invasion as a staging criterion

## Conclusion

This study challenges the notion that any PGM invasion should upstage OPSCC to T4. Invasion of the oropharyngeal portion of the PGM, given its superficial location close to the mucosal surface, appears to carry a prognosis more akin to early‐stage disease rather than advanced T3‐T4 tumors, although our study may be underpowered to detect small differences. Modifications to staging conventions may be warranted to better capture these nuances and guide appropriate treatment. Larger, multi‐institutional studies are needed to confirm these findings and further refine our understanding of PGM involvement in OPSCC prognosis and staging.

## Author Contributions

**Giancarlo Tirelli**, concept and design, analysis and interpretation of data, drafting of the manuscript supervision; **Nicoletta Gardenal**, concept and design, analysis and interpretation of data, drafting of the manuscript, supervision; **Jerry Polesel**, statistical analysis, analysis and interpretation of data, drafting of the manuscript; **Jasmina De Groodt**, acquisition, analysis and interpretation of data; **Erik Radin**, acquisition, analysis and interpretation of data; **Fabiola Giudici**, statistical analysis, analysis and interpretation of data; **Laura Iandolo**, acquisition, analysis and interpretation of data; **Simone Zucchini**, acquisition, analysis and interpretation of data; **Egidio Sia**, acquisition, analysis and interpretation of data; **Paolo Boscolo‐Rizzo**, Concept and design, analysis and interpretation of data, drafting of the manuscript, supervision.

## Disclosures

### Competing interests

None to declare.

### Funding source

The work of Drs Giudici and Polesel was partialy funded by Italian Ministry of Health "Ricerca Corrente" (no grant number).

## Supporting information

Supporting information.

Supporting information.
