# Supplementary figures and images for: Palatoglossus Muscle and T4 Category in the Eighth Edition of TNM Staging System for OPSCC
Source: Otolaryngol Head Neck Surg. 2024 Aug 27;171(6):1792–7. doi: 10.1002/ohn.957 (PMC11605017; doi:10.1002/ohn.957)

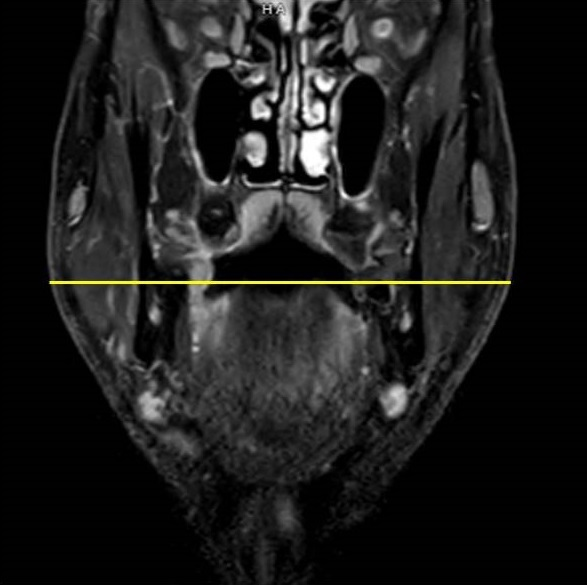

Supplement: Supplementary file 1 — Supporting information. [file OHN-171-1792-s002.tif]
