# Supplementary material for: Palatoglossus Muscle and T4 Category in the Eighth Edition of TNM Staging System for OPSCC
Source: Otolaryngol Head Neck Surg. 2024 Aug 27;171(6):1792–7. doi: 10.1002/ohn.957 (PMC11605017; doi:10.1002/ohn.957)
Supplement: Supplementary file 2 — Supporting information. [file OHN-171-1792-s001.docx]

Supplementary Figure. Coronal MRI scan showing the division of the palatoglossus muscle into oropharyngeal and oral portions. The division is made by an imaginary line extending through the dorsum at the base of the tongue. This delineation was used to assess the presence of palatoglossus invasion in patients with OPSCC.
